# Differential effects of angiotensin II type I receptor blockers on reducing intraocular pressure and TGFβ signaling in the mouse retina

**DOI:** 10.1371/journal.pone.0201719

**Published:** 2018-08-09

**Authors:** Ralph J. Hazlewood, Qingxia Chen, Frances K. Clark, John Kuchtey, Rachel W. Kuchtey

**Affiliations:** 1 Department of Ophthalmology and Visual Sciences, Vanderbilt University Medical Center, Nashville, TN, United States of America; 2 Department of Biostatistics, Vanderbilt University, Nashville, TN, United States of America; 3 Department of Molecular Physiology and Biophysics, Vanderbilt University, Nashville, TN, United States of America; Bascom Palmer Eye Institute, UNITED STATES

## Abstract

**Purpose:**

Angiotensin II type 1 receptor blockers (ARBs) have been investigated for their neuroprotective and intraocular pressure (IOP) lowering effects in treating glaucoma, but the reports have been inconsistent possibly because different compounds and models have been used. Here we selected three ARBs for head-to-head comparisons of their effects on IOP and transforming growth factor β (TGFβ) signaling, which is believed to play an important role in glaucoma pathogenesis.

**Methods:**

Three ARBs (losartan, irbesartan or telmisartan) or vehicle controls were administered *via* chow to C57BL/6J mice for up to 7 days. Drug concentrations in the eye, brain, and plasma were evaluated by liquid chromatography mass spectrometry. Cohorts of mice were randomly assigned to different treatments. IOP and blood pressure were measured before and after ARB treatment. Effects of ARBs on TGFβ signaling in the retina were evaluated by phosphorylated Smad2 (pSmad2) immunohistochemistry.

**Results:**

Physiologically relevant concentrations of losartan, irbesartan and telmisartan were detected in eye, brain and plasma after drug administration (n = 11 mice/treatment). Blood pressure was significantly reduced by all ARBs compared to vehicle-fed controls (all p-values < 0.001, n = 8–15 mice/treatment). Compared to vehicle control, IOP was significantly reduced by irbesartan (p = 0.030) and telmisartan (p = 0.019), but not by losartan (n = 14–17 mice/treatment). Constitutive pSmad2 fluorescence observed in retinal ganglion cells was significantly reduced by telmisartan (p = 0.034), but not by losartan or irbesartan (n = 3–4 mice/treatment).

**Conclusions:**

Administration *via* chow is an effective delivery method for ARBs, as evidenced by lowered blood pressure. ARBs vary in their abilities to lower IOP or reduce TGFβ signaling. Considering the significant roles of IOP and TGFβ in glaucoma pathogenesis, specific ARBs with dual effects, such as telmisartan, may be more effective than other ARBs for treating glaucoma.

## Introduction

Glaucoma is a neurodegenerative disorder and the leading cause of irreversible blindness worldwide [1]. Characteristic features are progressive loss of retina ganglion cells (RGCs) and their axons, resulting in visual deficits that can progress to blindness. Elevated intraocular pressure (IOP) is an important risk factor for glaucoma. Reducing IOP by surgical or pharmacological intervention remains the only treatment for glaucoma to date. However, for some patients, IOP is difficult to control and many patients continue to progress despite adequate IOP reduction, indicating that additional treatment modalities such as neuroprotection are needed.

Angiotensin II type I receptor blockers (ARBs) are a group of non-peptide competitive antagonists of the angiotensin II type I receptor (AT1R) [2]. ARBs inhibit both ligand-mediated activation by angiotensin II and ligand-independent stretch activation of the AT1R [3]. Following the first ARB, losartan, 7 additional ARBs (azilsartan, candesartan, eprosartan, irbesartan, olmesartan, telmisartan and valsartan) were developed. These compounds have some structural similarity, including a biphenyl moiety (except for telmisartan and eprosartan), with an attached acidic tetrazole group for losartan, olmesartan, valsartan, irbesartan and candesartan.

The AT1R is a primary mediator of the renin angiotensin system (RAS), in which systemic RAS regulates blood pressure and renal function. Many tissues, including the eye, express a localized RAS, which although less defined, serves multiple physiological roles including tissue remodeling and inflammation [4]. Activation of AT1R by angiotensin II or by mechanical stretch initiates multiple signal transduction cascades [5]. Blocking AT1R activation with ARBs has been proven highly effective in treating systemic hypertension, heart failure and kidney disease with minimal side effects.

Possible roles for RAS in ophthalmic diseases have been considered extensively [6, 7]. Specifically for glaucoma, RAS components, including the AT1R, have been identified in tissues relevant to glaucoma such as the ciliary body [8], neural retina and optic nerve [9–11]. Losartan has been shown to lower IOP in humans with normal or elevated IOP [12]. Similarly, olmesartan has been shown to lower IOP in animal models with experimentally elevated IOP [13–15]. Independent of IOP-lowering, ARBs also have neuroprotective effects, specifically in the context of glaucoma. Candesartan has been shown to reduce loss of RGCs in a normal tension glaucoma model and in a rat model of induced IOP elevation [16, 17]. Losartan has also been shown to have a neuroprotective effect for RGCs in mouse eyes with elevated IOP [11].

With combined IOP-lowering and neuroprotective properties, ARBs are attractive candidates for treating glaucoma. In addition, ligand activation of the AT1R stimulates signal transduction through transforming growth factor beta (TGFβ) [18–21] and this activity is inhibited by ARBs [22–24]. Acting as inverse agonists, ARBs also inhibit ligand-independent stretch activation of the AT1R [3, 25]. TGFβ activity and stretch activation are particularly relevant since elevated TGFβ [26, 27] and altered mechanotransduction [28] likely contribute to glaucoma pathogenesis.

Because ARBs differ in their pharmacological properties, such as receptor binding affinity, receptor off-rates, and inverse agonism [2, 25], effectiveness in treating glaucoma could depend on which ARB is used. In this study, head-to-head comparisons of 3 ARBs (losartan, irbesartan and telmisartan) with divergent properties were made of their ability to lower IOP and reduce TGFβ signaling in the retina of normal mice. These abilities were found to vary depending on the ARB used, suggesting that investigations of these drugs as potential glaucoma treatments in regard to IOP and TGFβ control should take into account pharmacological variation of the different ARB members.

## Materials and methods

### Mice

All experiments were carried out in accordance with the Association for Research in Vision and Ophthalmology (ARVO) statement for the Use of Animals in Ophthalmic and Vision Research and were approved by the Vanderbilt University Institutional Animal Care and Use Committee. C57BL/6J mice purchased from the Jackson Laboratory were used in this study. Males at 3 months of age were used for all experiments, except for a preliminary experiment investigating IOP effects of losartan delivered *via* drinking water, which used 1-year old female C57BL/6J mice. Mice were housed in a facility managed by Vanderbilt University Division of Animal Care, with *ad libidum* access to water and standard mouse chow and a 12 h light cycle (lights on at 6:30 a.m. and off at 6:30 p.m.). At the end of experiments, mice were sacrificed by carbon dioxide inhalation.

### Drugs

Losartan potassium, irbesartan and telmisartan, all with > 98% purity, were obtained from AK Scientific (Union City, CA, USA). Losartan carboxylic acid (EXP 3174, 95% purity) was obtained from Santa Cruz Biotechnology (sc-218661, Santa Cruz, CA, USA). Mouse chow containing losartan, irbesartan or telmisartan from the above sources was formulated by Envigo Tekland Diets (Madison, WI, USA) at a concentration of 2 g/kg in 5001 base diet. This dose was chosen based on previous reports that show a reduction in TGFβ signaling in the aorta of mice with Marfan syndrome [23]. Mice receiving normal chow were fed 5001 base diet without additions (Envigo Teklad Diets). To determine the rate of chow consumption, an amount of chow was weighed and placed in the feed holder of cages housing 3–4 mice, and after 3 days, the remaining chow was weighed.

### IOP measurement

IOP of mice anesthetized with 2.5% isoflurane in oxygen delivered at 1.5 L/min by an inhalation anesthesia system (Vet Equip, Livermore, CA, USA) was measured using a Tonolab rebound tonometer (Icare, Vantaa, Finland) following manufacturer’s recommendations by an operator blinded to the treatment status of the mice. Measurements were performed within 3 mins of immobilization to minimize isoflurane-induced IOP changes [29] and at the same time of day (1:00–3:00 p.m.) to avoid diurnal variation [30]. A total of 64 mice were used for these experiments with n = 17 for losartan and irbesartan treatment, n = 16 for telmisartan treatment and n = 14 for normal chow control.

### BP measurement

BP was measured by the tail-cuff method (BP 2000 Blood Pressure Analysis System, Visitech Systems, Apex, NC, USA) according to manufacturer recommendations and as previously described [31, 32] by an operator blinded to the treatment status of the mice. In brief, mice were restrained and their tail placed through an inflatable tail cuff held in place by adhesive tape. Systolic and diastolic BP was determined as the mean of readings from 20 cycles of inflation-deflation of the tail-cuff, which were preceded by 10 acclimation measurements. Mice were conditioned to the system prior to experimental measurements by being subjected to the full recording procedure daily for several days. Acclimation and BP measurements were performed between 9:00 and 11:00 a.m. Since diastolic measurements are inherently inaccurate with this method [33, 34], only systolic BP is reported here. A total of 53 mice were used for these experiments with n = 15 for losartan, irbesartan and telmisartan treatment and n = 8 for normal chow control.

### Tissue sample processing for liquid chromatography mass spectrometry

Mice housed 3–4/cage were supplied with drinking water containing 1.2 g/L losartan in place of their normal water or 2 g/kg chow containing irbesartan, telmisartan, or losartan in place of normal chow. For losartan drinking water experiments, on the third day of treatment, one set of mice was sacrificed at 9:00 a.m., near the end of the active nocturnal phase, to approximate peak drug concentrations during the animal’s active phase and another set sacrificed at 5:30 p.m. to approximate trough concentrations during the animal’s inactive phase. For the chow delivery experiments, mice were sacrificed on the morning of the third day of treatment. Following euthanasia, whole blood was collected into EDTA-treated tubes by cardiac puncture and eyes were enucleated and placed in 2 ml polypropylene tubes. Plasma was separated from whole blood by centrifugation for 15 min at 3000 x g, transferred to polypropylene tubes and stored at -20°C. Cortex (brain) was carefully dissected from the skull and placed in 2 mL polypropylene tubes. Enucleated eyes and brain were immediately flash frozen on a dry ice/ethanol bath and stored at -80°C. Samples were allowed to thaw on ice for further processing. Aliquots of 100 μL plasma were transferred to clean tubes, to which deuterated losartan (losartan-d3, Toronto Research Chemicals, Toronto, Ont., CA) was added as internal control. Plasma samples with internal control were de-proteinated by addition of 300 μL acetonitrile, centrifugation at 10,000 x g for 5 min at 4°C and transfer of supernatant to clean tubes. Plasma supernatant was evaporated under a gentle stream of N_2_ gas at 25°C and reconstituted in 100 μL of 1:1 acetonitrile:water. Upon thaw, brain and both eyes from each mouse were minced in 400 μL methanol to which deuterated losartan was added as internal control. For eye and brain samples, 40 μL of tissue extract containing internal control was added to a fresh tube, to which 60 μL normal mouse plasma was added to normalize for tissue matrix effects [35, 36]. Extracts diluted with plasma were de-proteinated and evaporated as described above and reconstituted in 100 μL 1:1 acetonitrile:water. Calibration standards of 5, 10, 50, 100 and 1000 nM losartan and EXP3174 with deuterated losartan as internal control were prepared in 100 μL normal mouse plasma, de-proteinated, evaporated and re-suspended as described above. For experiments involving chow, aliquots of tissue extracts were transferred to clean microcentrifuge tubes and spiked with the appropriate working stocks of losartan, EXP3174, irbesartan, and telmisartan combined solution and internal standard working stock of deuterated isoforms of each drug compound. Spiked samples were vortexed for 5 min on ice then sonicated for 2 min on ice. The resulting homogenate was centrifuged for 10 min at 3000 x g and supernatant removed to a clean, polypropylene microcentrifuge tube. Spiked plasma samples were lightly vortexed and deproteinated with 600 μL HPLC-grade acetonitrile. Precipitated proteins were removed by centrifugation (10,000 x *g*, 10 min at 4°C). The clear supernatant (~800 μL) of each plasma sample was transferred to a clean Eppendorf tube and all tissue samples were evaporated under a gentle stream of N_2_ gas at 25°C. The residue was then reconstituted in mobile phase in 100 μL of methanol/water (1:1), vigorously vortexed, and transferred to 200 μL silanized autosample vials equipped with Teflon-lined bonded rubber septa.

### Liquid chromatography mass spectrometry (LC/MS)

Sample analyses were carried out at the Mass Spectrometry Core at Vanderbilt University using a Waters Acquity UPLC system (Waters, Milford, MA, USA), made up of a binary solvent manager, refrigerated sample manager and a heated column manager. Tandem mass spectrometric detection was performed using a TSQ Quantum triple quadrupole mass spectrometer (Thermo Scientific, San Jose, CA, USA). An XTerra MS C18 analytical column (2.1 mm x 100 mm, 3.5 μm particle size, Waters) was used for all chromatographic separations. Mobile phases were made up of 0.2% HCOOH in (A) H_2_O/CH_3_CN (95:5) and (B) H_2_O/CH_3_CN (5:95). Gradient conditions were as follows: 0–1 min, B = 5%; 1–8 min, B = 5–100%; 8–10 min, B = 100%; 10–10.5 min, B = 100–5%; 10.5–15 min, B = 5%. The flow rate was maintained at 300 μL/min. Quantitation was based on multiple reaction monitoring detection in positive ion mode. Data acquisition and quantitative spectral analysis were done using Xcalibur version 2.0.7 SP1 and LCQuan version 2.7, respectively. Injection volumes of 10 μL of prepared plasma, eye and standard samples were applied to the column. Calibration curves were constructed by plotting peak area ratios (analyte:internal control) against analyte concentrations. A weighting factor of 1/C_t_^2^ was applied in the linear least-squares regression analysis to maintain homogeneity of variance across the concentration range. A total of 33 mice were used for determining drug concentrations after chow administration, with n = 11 for losartan, irbesartan and telmisartan.

### Immunohistochemistry

Mice treated for 3 days with chow containing either losartan (n = 3), irbesartan (n = 4) or telmisartan (n = 4), or normal chow (n = 4) were euthanized then cardiac perfused with 20 mL PBS followed by 20 mL PBS/4% paraformaldehyde (PFA). Eyes were enucleated and post-fixed in PBS/PFA for an additional 24–48 h and embedded in paraffin. Five micron-thick sections were deparaffinized, incubated at 90°C for 30 mins in 10 mM sodium citrate buffer, pH 6.0 for epitope retrieval and then incubated for 2 h at room temperature in blocking solution (PBS/5% normal donkey serum/1% BSA/0.1% Tween-20). Sections were then incubated with rabbit polyclonal antibody to pSmad2 (1:100, Thermo Fisher Scientific, MA, USA) in blocking solution overnight at 4°C in a humidified chamber. Following three 5 min washes in wash solution (PBS/0.1% Tween-20), sections were incubated in AlexaFluor-546 labeled donkey anti-rabbit IgG diluted 1:1000 in blocking solution for 2 h at room temperature in a dark humidified chamber. Sections were washed again three times for 5 mins each in wash solution and cover-slipped with Prolong Gold Antifade with DAPI mounting medium (Thermo Fisher Scientific). Sections were imaged using a Nikon AZ 100M upright, wide-field microscope using a 5x Plan Fluor/0.5 NA air objective and captured with a Nikon DS-Ri1 color camera (Nikon Instruments, Melville, NY, USA). Retinal images were acquired adjacent to the optic nerve head. Immunostaining and imaging experiments using the right eye of each mouse were carried out in batches of sections that included samples from two vehicle-fed mice and two samples each from losartan, irbesartan and telmisartan-fed mice using identical image acquisition parameters within each batch. Immunostaining and imaging experiments were carried out twice for each batch of samples.

Mean fluorescence intensity of pSmad2 immunofluorescence was quantified using FIJI (ImageJ) biological image analysis package [37]. Background-subtracted mean fluorescence intensity of the RGC layer was determined by subtracting the mean background intensity within the adjacent inner plexiform layer from the mean fluorescence intensity within a mask drawn around the RGC layer. Normalized background-subtracted mean fluorescence intensity within each batch of samples was determined by dividing the background-subtracted mean fluorescence intensity of each sample by the average of the background-subtracted mean fluorescence intensities of the two vehicle-fed control samples. For each mouse, normalized background-subtracted mean fluorescence intensity was determined as the average of duplicate experiments and is presented as normalized mean fluorescence.

### Statistical analysis

Sample sizes were not pre-determined by statistical methods, but are similar to or exceed numbers typical of similar experiments[16, 17]. The IOP changes of treated mice relative to the IOP changes of normal chow fed mice were presented with mean and 95% confidence interval based on t-statistics. A linear regression model for BP change from baseline was used to study effects of ARBs on BP, adjusting for baseline BP. For comparing IOP in groups of mice untreated or treated with ARBs for 3 and 7 days, a mixed effects model that included baseline IOP, left *versus* right eye, time of measurement and interaction between time and drug with random effects of intercept and slope within individual mice was used, adjusting for correlation between left and right eye within the same mouse, as well as repeated measurements over time. A one-way ANOVA was used for comparing chow pSmad2 fluorescence of mice untreated or treated with ARBs. The models for BP, IOP, and pSmad2 fluorescence were followed by Dunnett’s method for multiple comparisons correction. Differences in the change in IOP for each drug, relative to change in IOP of normal-fed control were evaluated at day 3 and day 7 of treatment, and differences in the rate of change in IOP were evaluated as the slope of the line defined by relative change at days 3 and 7. Corrected p-values < 0.05 were considered significant. Linear regression and mixed effect model analysis was performed with R 3.4.1 (R Core Team. R: A language and environment for statistical computing. R Foundation for Statistical Computing, Vienna, Austria. URL http://www.R-project.org/, 2013). All other statistical analysis was performed using GraphPad Prism version 7.00 for Windows software (GraphPad Software, La Jolla, CA, USA).

## Results

### Effect of losartan delivered by drinking water

**Fig 1:**
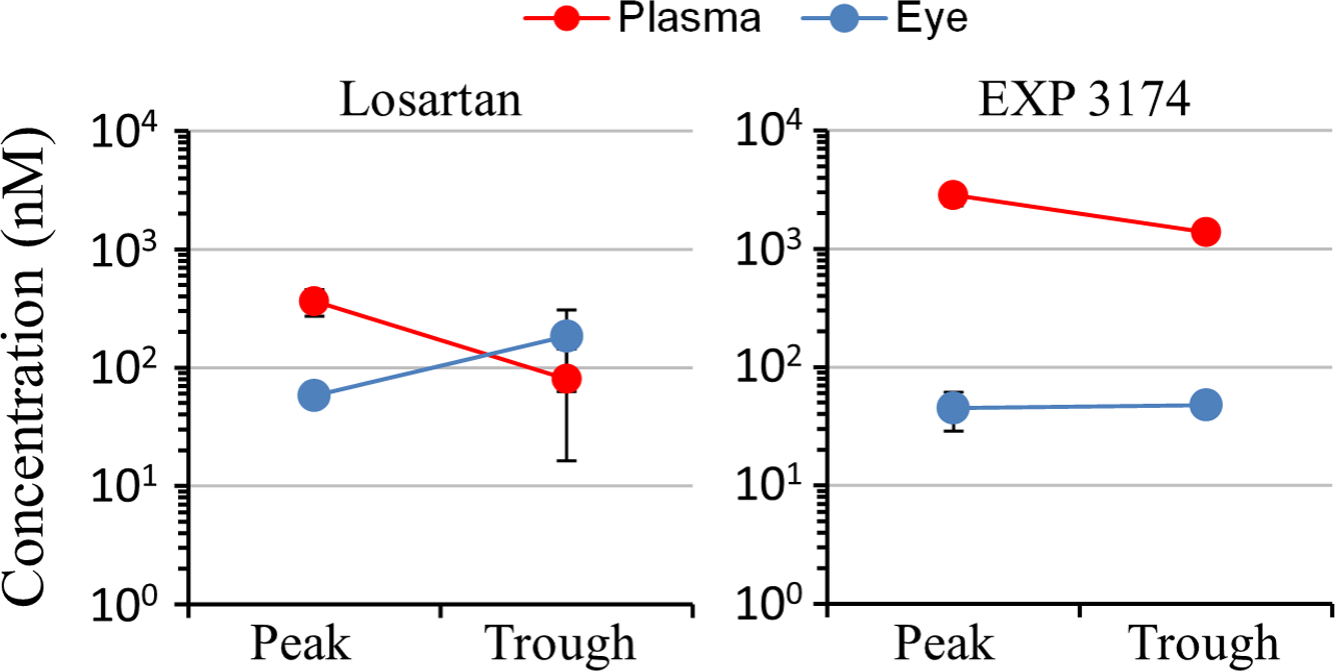
Tissue distribution of losartan after delivery *via* drinking water. Concentrations of losartan (left) and its active metabolite, EXP 3174 (right) in the plasma (red symbols) and eye (blue symbols) were determined by LC/MS after 3 days of treatment with 1.2 g/L losartan in water available *ad libidum*. At different time points, mice (n = 3) were sacrificed and eyes and plasma were collected for LC/MS. Approximate peak concentrations were determined by gathering samples at the end of the active nocturnal phase. Approximate trough concentrations were determined by gathering samples at the end of the inactive daytime phase. Symbols represent mean/SD.

To determine drug distribution, 3-month-old male C57BL/6J mice were treated with 1.2 g/L losartan in drinking water. After 3 days of treatment, mice were sacrificed in the morning, following their nocturnal active phase, which should represent approximate peak concentrations and at the end of the inactive daytime phase, to approximate trough concentrations. Plasma and eyes were harvested to determine the concentrations of losartan and its active metabolite, EXP 3174. LC/MS analysis revealed that the peak and trough levels of losartan and EXP 3174, were similar (Fig 1), indicating that this dosing method resulted in stable tissue concentrations. Importantly, losartan and EXP3174 were detected in the eye tissue, as required for direct ocular effects.

In a preliminary experiment, the effect on IOP of 1.2 g/L losartan delivered by drinking water was investigated in a set of 10 female C57BL/6 mice 1 year of age. Assuming a daily water consumption rate of 8 mL/30 g body weight [38], mice would receive a losartan dose of 320 mg/kg/day. On the morning of the first day of the experiment, baseline IOP was determined in the right eye of each mouse and delivery of losartan in drinking water was initiated. On the morning of the third day of treatment, IOP was again determined. Treatment of mice for 3 days with losartan did not affect IOP (14.8 +/-1.4 mmHg before, 15.4 +/- 1.9 mmHg after losartan treatment, mean +/-SD, p = 0.4, data not shown). These results suggest that losartan does not lower IOP in normal mice.

### Tissue concentrations of losartan, irbesartan, and telmisartan delivered by chow

Since delivery of losartan by drinking water resulted in substantial eye concentrations, attempts were made to solubilize irbesartan and telmisartan in unbuffered water. Unlike losartan, they readily precipitated out of solution. Therefore, the drugs were incorporated into solid chow at a concentration of 2 g drug/kg chow, which, assuming normal daily consumption of chow of 4.5 g/30 g body weight [38], would deliver a dose of 300 mg/kg/day, similar to the dose given by losartan in drinking water. Because the drugs could change the taste of the chow and result in aversion to eating, the rate of consumption was monitored by weighing chow at the beginning and end of a 3 day administration period. There were no significant differences between groups in the amount of chow consumed (all p-values > 0.5), which was approximately 4 g mouse/day (data not shown), as expected for C57BL/6 mice [38]. These results indicate no strong aversion to eating ARB-containing chow, which delivered a dose of approximately 280 mg/kg/day.

**Fig 2:**
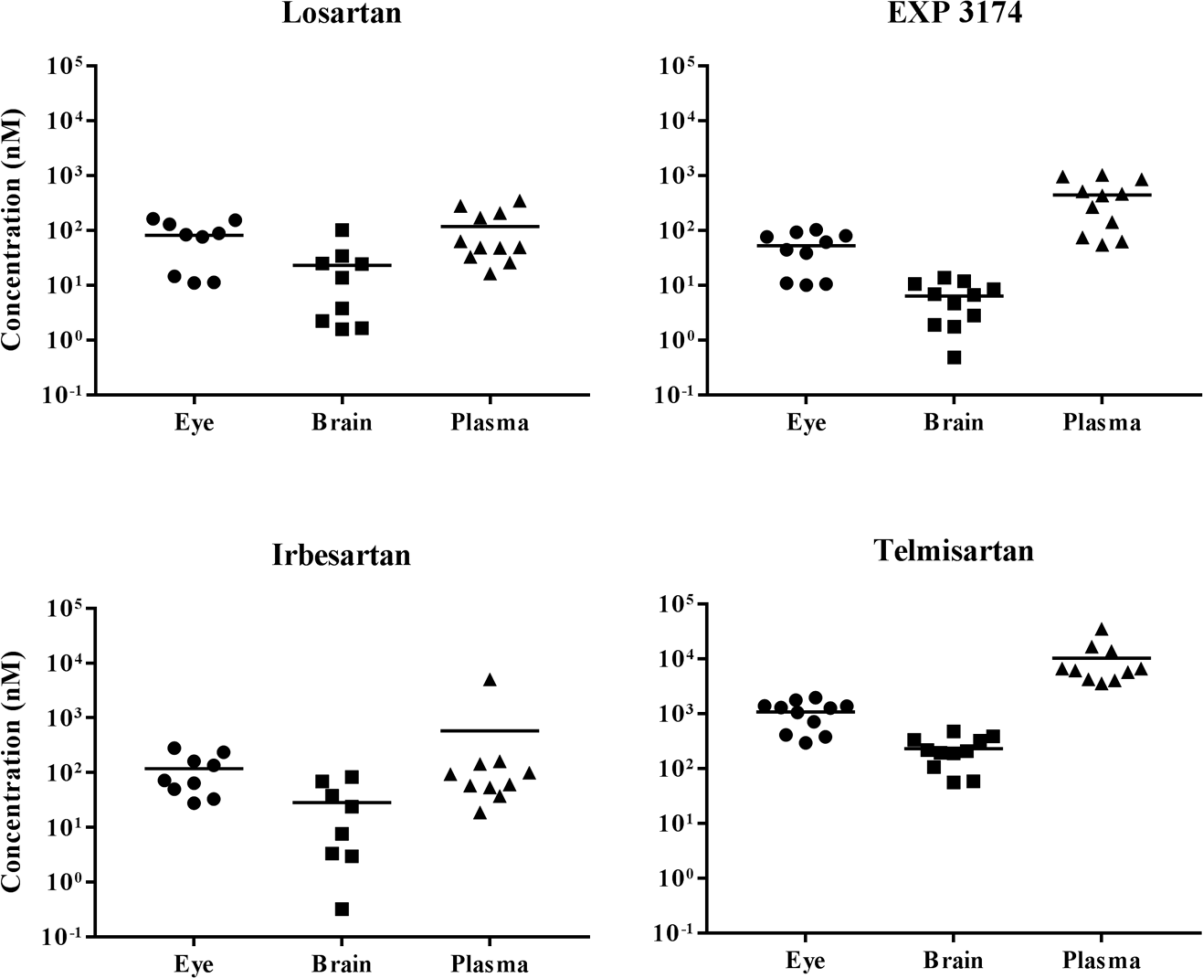
Tissue distribution of ARBs after delivery *via* chow. Mice (n = 11) were fed chow containing losartan, irbesartan or telmisartan available *ad libidum*. After 3 days, mice were sacrificed and eyes, brain, and plasma were collected for LC/MS analysis of drug concentrations. Irbesartan, telmisartan, losartan and EXP 3174 were detected in eyes, brain, and plasma samples. Solid symbols represent data from individual mice, with mean indicated as horizontal line for each group. Data are visualized on logarithmic scale.

After 3 days administration of losartan, irbesartan, or telmisartan *via* chow, mice were sacrificed and tissue extracts made from plasma, eyes and brain for determination of drug concentration by LC/MS (Fig 2). Losartan and EXP 3174 were detected in the eye and plasma at similar concentrations as for losartan delivered in drinking water, indicating effectiveness of dosing *via* chow. Moreover, losartan and EXP 3174 were also detected in the brain, indicating ability of the drug to cross the blood-brain barrier. Similar concentrations of irbesartan were also detected in the eye, brain and plasma. Telmisartan achieved approximately 10-fold higher concentrations as compared to losartan and irbesartan, likely due to its higher lipophilicity and volume of distribution. All three ARBs appear to have the ability to cross the blood-brain barrier, perhaps indicating an ability to cross the blood retinal barriers as well.

### Effects of losartan, irbesartan and telmisartan on BP and IOP

**Fig 3:**
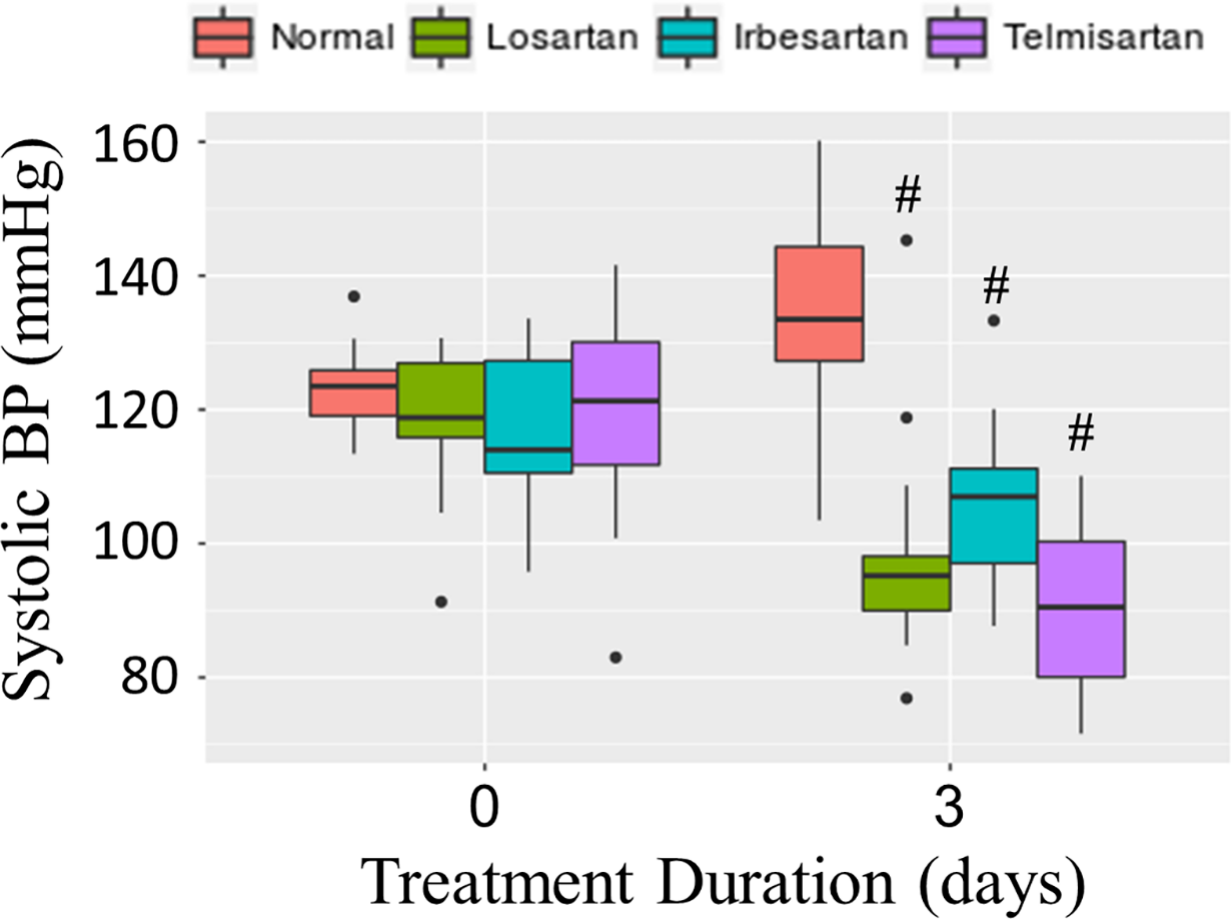
Systolic BP before and after treatment with ARBs for 3 days. Systolic BP was measured before (day 0) and after (day 3) feeding mice chow containing losartan (n = 15, green), irbesartan (n = 15, blue) or telmisartan (n = 15, violet) at 2 g drug/kg chow, or normal chow (n = 8, orange) available *ad libidum*. Compared to mice fed normal chow, mice fed losartan, irbesartan or telmisartan chow had significantly lower BP (p < 10^−4^). Box plots show the median (thick line), first and third quatriles (lower and upper box sides), with vertical lines representing 5^th^ and 95^th^ percentiles. Data outside the 5^th^ to 95^th^ percentiles are shown as individual data points (black symbols).

To investigate whether the dose of ARBs from chow administration was physiologically effective, BP was measured in mice using the tail-cuff method before and after ARB treatment, as ARBs are established BP-lowering agents. As shown in Fig 3, all ARBs tested lowered systolic BP after 3 days of treatment from an average baseline of 119 mmHg. Compared to the normal control group, BP was significantly reduced by all ARBs, adjusting for baseline BP (all p-values < 0.001). These results indicate that delivery of ARBs *via* chow resulted in physiologically effective doses.

**Fig 4:**
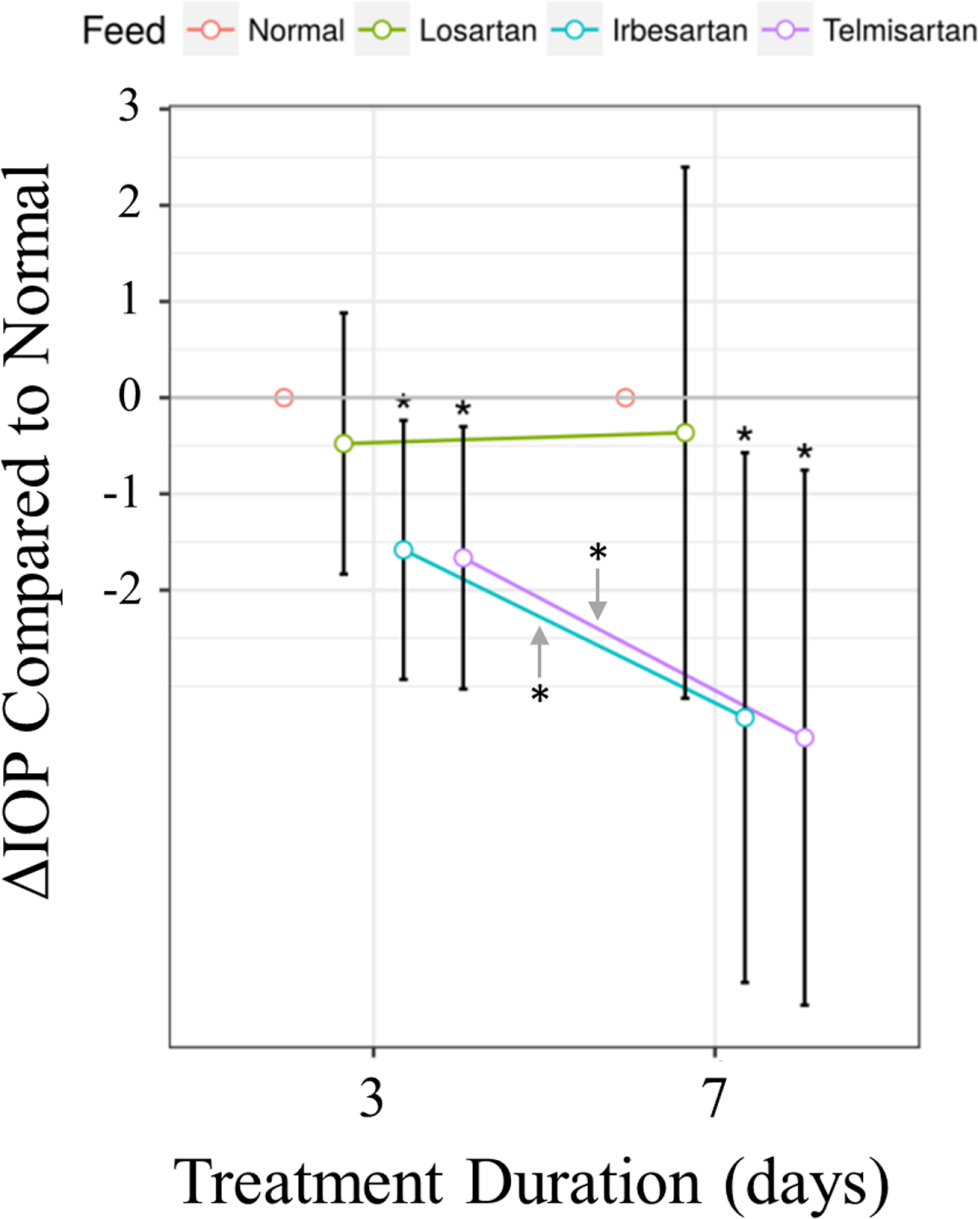
Change in IOP after treatment with ARBs for 3 and 7 days. Significant reductions of IOP were found for mice treated with irbesartan (p = 0.016 and 0.013) and telmisartan (p = 0.012 and 0.008) at day 3 and 7, respectively, compared to mice fed normal chow, but not for losartan treated mice. IOP decreased significantly faster for mice treated with irbesartan (p = 0.030) and telmisartan (p = 0.019), compared to mice fed normal chow, while losartan had no significant effect. Symbols and error bars represent mean and 95% confidence intervals; orange: normal chow, green: losartan, blue: irbesartan, purple: telmisartan. Data are from 14–17 mice/treatment for days 0 and 3 and 10 mice/treatment for day 7. Median IOP values for day 0 were 17.2, 16.9, 17.0 and 16.7 for normal, losartan, irbesartan and telmisartan-treated mice, respectively.

To test for effects of ARBs on IOP, groups of mice were fed with either normal chow or chow containing losartan, irbesartan or telmisartan. IOP was measured in both eyes of each mouse (n = 64) before and 3 days after initiating ARB treatment. For some mice (n = 40), IOPs were additionally measured after 7 days of ARB treatment. As shown in Fig 4, significant decreases in IOP compared to normal chow control was found for irbesartan (p = 0.016 and 0.013) and telmisartan (p = 0.012 and 0.008) after 3 and 7 days of treatment, respectively, but not for losartan treated mice. In addition, the rate of IOP reduction was significantly greater than control for irbesartan (p = 0.030) and telmisartan (p = 0.019), but not for losartan-treated mice. These results, that irbesartan and telmisartan lower IOP but losartan does not, are consistent with the hypothesis that physiological effects can vary significantly between ARBs.

### Decreased pSmad2 in the RGC layer of telmisartan-treated mice

**Fig 5:**
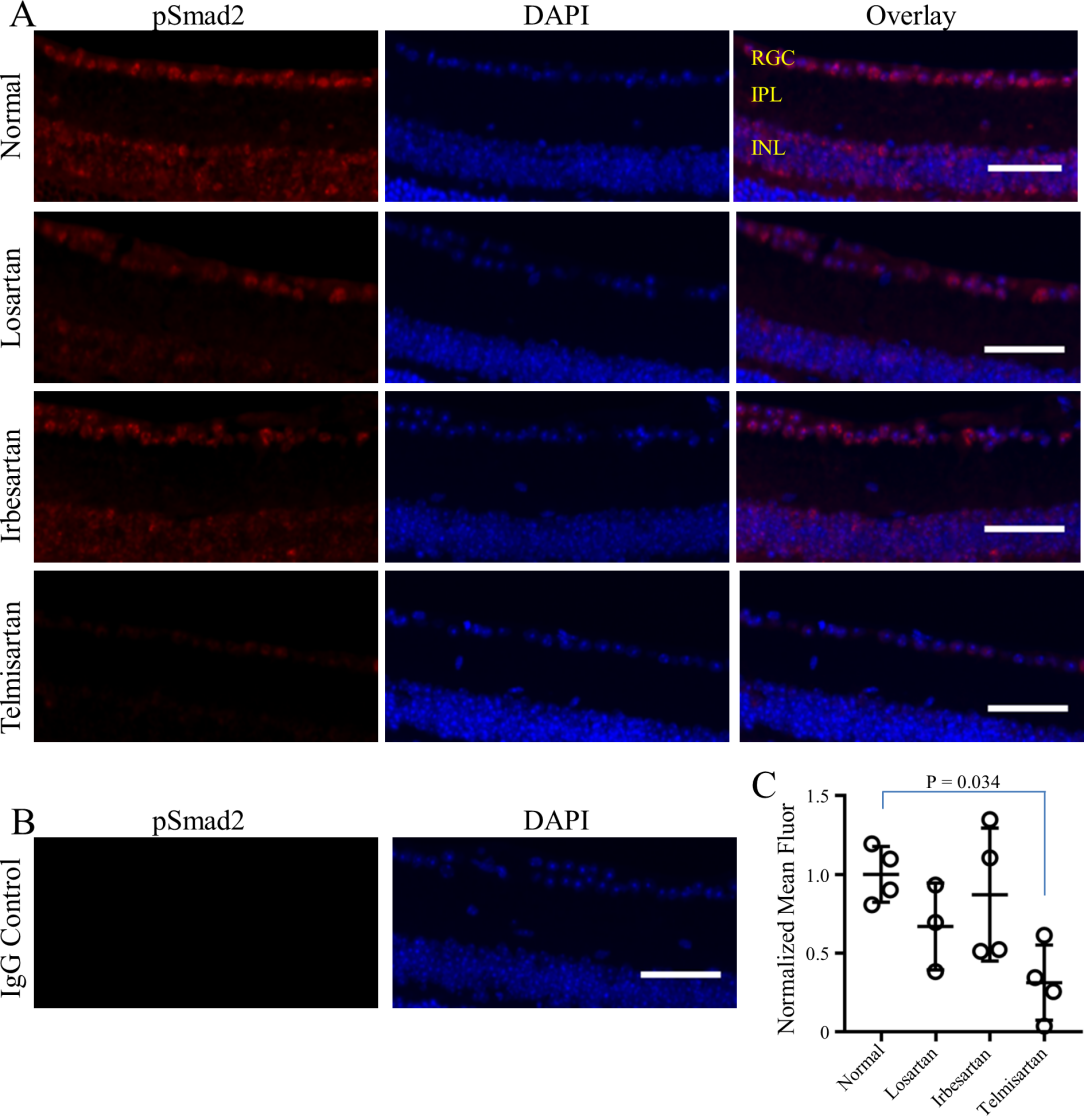
Reduced TGFβ signal transduction in the RGC layer of ARB-treated mice. (A) Representative immunostaining for pSmad2 (red, left column) and DAPI-staining of cell nuclei (blue, middle column) shows nuclear pSmad2 in overlay images (pink, right column). Pattern indicates constitutive TGFβ signal transduction in the inner nuclear and RGC layers of mice fed normal chow (upper row) that is reduced in mice fed ARB-containing chows (lower three rows), most strongly by telmisartan (lower row). IgG negative control (B) shows lack of non-specific staining. Quantification of pSmad2 fluorescence (red) in the RGC layer (C) shows statistically significant reduction in telmisartan-treated mice, with a 70% reduction compared to normal fed mice (p = 0.034). Results are from duplicate experiments from one eye of each individual mouse; n = 4 for normal, irbesartan and telmisartan; n = 3 for losartan. Symbols represent the average pSmad2 red fluorescence for each mouse with mean/SD for each treatment shown in (C). Retinal layers are indicated (upper right panel, A): RGC = RGC layer; IPL = inner plexiform layer; INL = inner nuclear layer. Scale bars = 50 μm.

Immunohistochemistry of pSmad2 is a read-out for active TGFβ signaling, which results in phosphorylation and nuclear translocation of Smad2. To investigate the effect of ARBs on TGFβ signaling in the eye, pSmad2 immunohistochemistry was performed on sagittal sections of eyes from mice fed normal or ARB-containing chow for 7 days. In the retina of mice treated with normal chow, nuclear pSmad2 fluorescence (red) was observed in the inner nuclear layer and, most prominently, in the RGC layer (Fig 5A, top row), indicating constitutive TGFβ signaling in the inner retina of normal mice. In mice treated with telmisartan, pSmad2 fluorescence of the RGC layer was significantly reduced (p = 0.034, Fig 5C), while losartan and irbesartan had no significant effect. These findings further support heterogeneity in the effects of different ARBs, with telmisartan being highly effective at attenuating TGFβ signaling compared to losartan and irbesartan. Furthermore, these findings indicate that ARBs may have the ability to cross the blood-retinal barrier where they can directly act on RGCs.

## Discussion

The present study is significant in its head-to-head comparisons of several ARBs. Previous studies focused on the effects of single ARBs on IOP and/or neuroprotection of RGCs [11–13, 15–17] and were largely unique in the choice of dose, route of administration, species, glaucoma model and specific ARB used, resulting in somewhat conflicting results and difficulty for comparison.

Initial investigations with the parent ARB losartan, were promising, as they suggested favorable IOP-lowering properties. In a study by Costagliola et al. [12], a 50 mg oral dose of losartan reduced IOP in human glaucoma patients with elevated IOP, and in normal controls, with a mean reduction of as much as 16%. Similarly, in rabbit eyes with induced IOP elevation, Shah et al. [14] showed IOP-lowering effects of eye drops consisting of 0.1% losartan in saline. However, in a study with CD1 mice by Quigley et al., 0.6 g/L losartan delivered *via* drinking water showed no effect on IOP in normotensive eyes or in eyes with elevated IOP induced by microbeads injection [11]. Consistent with this, we found no effect on IOP of losartan delivered at higher doses with 1.2 g/L in drinking water (data not shown) or 2 g/kg in chow (Fig 4) in normal C57BL/6J mice.

In a study with another ARB, candesartan, Semba et al. [16] found no effect on IOP of 10 mg/kg/day of candesartan delivered by oral gavage in a mouse model of normotensive glaucoma. However, significant protections against loss of RGCs, thinning of the ganglion cell complex and reduced responses in multifocal electroretinograms were reported [16]. Conversely, olmesartan was shown to have IOP-lowering effects in rabbits with experimental and inherited elevated IOP [13], and in monkeys with laser-induced IOP elevation [15]. Despite these promising IOP effects, a phase II clinical trial for topical olmesartan sponsored by Santen Pharmaceutical and Daiichi Sankyo Company was terminated in 2008 due to insufficient magnitude and lack of clear dose-response relationship [39].

The contradictory results of previous studies could in part be due to differences in efficacies of the different ARB family members used. In our study, incorporation of ARBs in chow allowed for direct comparisons between losartan, irbesartan and telmisartan using identical delivery methods. We chose an ARB dose that is in the higher range of doses of losartan previously shown to reduce TGFβ in mice. Delivery of 0.6 g/L and 0.9 g/L losartan in drinking water has been shown to reduce TGFβ signaling in the aorta in a mouse model of Marfan syndrome [23, 40] and in skeletal muscle of mice with induced injury, respectively [41]. Further reduction of circulating levels of TGFβ in the mouse Marfan model has been shown for 1.2 g/L, as compared to 0.6 g/L losartan in drinking water [42]. To obtain maximal effect on TGFβ signaling, we used 1.2 g/L in drinking water and incorporated losartan, irbesartan and telmisartan into chow at 2 g/kg, which resulted in a similar dose. In our study, the animals ate normal amounts of drug-containing chow (data not shown) and BP reduction was moderate (Fig 3.), suggesting the ARBs dosage was well tolerated and did not have overt toxic effects. Similarly, we did not observe any adverse events during the treatment period and examination of kidneys and heart after ARB treatment were unremarkable (data not shown).

Another unique aspect of our study is that we measured ocular concentrations of ARBs. We found significant concentrations of losartan and its metabolite EXP 3174, irbesartan, and telmisartan in eye tissue (Figs 1 and 2.), which is a requirement for direct ocular effects. We also found appreciable concentrations of ARBs in the brain, demonstrating their ability to cross the blood-brain barrier (Fig 2). Furthermore, the reduction of TGFβ signaling observed in the RGC layer (Fig 5) suggests that ARBs can cross the blood-retinal-barrier to act directly on RGCs, supportive of neuroprotective effects found in animal models of glaucoma [11, 16, 17].

Differences between ARBs in bioavailability, tissue distribution, receptor affinity, inverse agonist activity and off-target effects could be clinically relevant [2, 43]. While the efficacy of different ARBs in lowering BP are likely similar [44], as seen in our study (Fig 3), there are known differences between ARBs in their efficacy for treating disorders such as diabetes, atrial fibrillation, myocardial infraction and stroke [45]. Our results show that there is variation between ARBs in their ability to reduce IOP or inhibit TGFβ signaling in the eye, two important features relevant to glaucoma. These differential capabilities further indicate that studies investigating the potential utility of ARBs in treating glaucoma should take into account which ARB is used, rather than assuming common effects for the entire class of drugs.

Our study is the first to investigate the effects of irbesartan and telmisartan on IOP, both of which were found to have significant IOP-lowering effects (Fig 4). Compared to day 0, irbesartan-treated mice had an average reduction of the median IOP of 15.4%. Telmisartan also significantly affected IOP, with an average reduction of the median IOP of 13.3%. In contrast, losartan did not significantly lower IOP. The results of our head-to-head comparisons of ARBs suggest that there are significant differences in their abilities to lower IOP.

Independent of IOP-lowering, ARBs also have neuroprotective effects, specifically in the context of glaucoma. In a mouse model of normal tension glaucoma, orally administered candesartan reduced loss of RGCs and thinning of the inner retina, without affecting IOP [16]. In a rat model with IOP elevation induced by episcleral vein cauterization, oral candesartan reduced RGC loss but did not affect IOP [17]. Recently, Quigley et al. showed that orally delivered losartan *via* drinking water had a neuroprotective effect for RGCs in mouse eyes with elevated IOP induced by microbead injection, though a similar effect was not seen in response to optic nerve crush [11].

Similar to a previous study by Braunger et al. [46], we found prominent pSmad2 immunofluorescence in the nuclei of cells in the inner nuclear and RGC layers of the retina, indicating constitutive TGFβ signal transduction. In our study, pSmad2 immunofluorescence in the RGC layer was significantly reduced in mice treated with telmisartan, but not in those treated with losartan or irbesartan (Fig 5). To the extent that elevated TGFβ is important for glaucoma pathogenesis [26, 27], reduced TGFβ signaling could be beneficial, particularly in the aqueous humor outflow pathway. In our samples, we found high variability of pSmad2 staining in the aqueous outflow pathway and could not make conclusions about the effects of ARBs in these structures. It should be noted that at least during development, TGFβ promotes survival of RGCs [46], indicating potential opposing effects of TGFβ suppression. However, the suppression of TGFβ signaling suggests that ARBs cross the blood-retinal barrier and interact directly with RGCs. Direct interaction is possible, since the AT1R is expressed in most retinal neurons, including in the nerve fibers of the ganglion cell layer [47]. Direct interaction of ARBs with RGCs could exert a neuroprotective effect, as has been reported for cultured neurons exposed to neurodegenerative stimuli and RGCs in retinal explant cultures [48–50].

Variation between ARBs in their pharmacological properties may result in differences in physiological effects [2, 43]. Irbesartan has the highest affinity for the AT1R, with a K_d_ of approximately 2 nM, compared to losartan which has the lowest affinity, with a K_d_ of approximately of 10 nM [43]. Telmisartan, one of the more divergent structures of the group, is the most lipophilic ARB with the greatest potential for distribution into tissues [2]. Comparisons were made between losartan, irbesartan and telmisartan, three ARBs with divergent properties, to test the hypothesis that individual ARBs can have different physiological effects. A striking finding was that tissue concentrations of telmisartan, including the eye, were an order of magnitude higher than the other ARBs. This is consistent with the lipophilicity of the compound which may facilitate penetration of the blood-retina and blood-aqueous humor barriers and achieve concentrations well above the k_d_ of the AT1R, and may explain effectiveness of telmisartan at both lowering IOP and interacting with RGCs.

The divergent properties of ARBs found in the present study were observed in normal eyes, and we predict divergent properties would also be found in glaucomatous eyes often associated with elevated IOP. Reduction in pathologically activated TGFβ signaling by ARBs has been demonstrated in disease states such as Marfan syndrome [23, 40, 42], muscle injury [41], autoimmune encephalitis [51], and renal injury [52], with significantly beneficial results. In glaucoma patients, elevated levels of TGFβ may play an important role in elevation of IOP and optic nerve injury by changing the extracellular matrix composition of the trabecular meshwork and lamina cribrosa [26, 27]. If TGFβ signaling is enhanced in glaucoma, reductions by ARBs could be larger in magnitude and more widespread than observed in normal mice, and could prove beneficial for glaucoma treatment. It should be noted that ARBs ability to attenuate TGFβ signaling has an additional potential benefit for glaucoma patients in preventing scar formation after trabeculectomy surgery [53].

In the present study, systemic delivery by the oral route was used. Glaucoma patients could be treated with oral doses of ARBs, a convenience possibly resulting in better patient compliance compared to eye drops. Alternatively, in a personalized medicine approach, patients with systemic hypertension and glaucoma could choose ARBs instead of, or in addition to, angiotensin converting enzyme inhibitors to treat hypertension while receiving additional benefits for treating glaucoma.

Delivery of ARBs by eye drops to achieve localized effective drug concentrations would likely result in relatively low systemic concentrations, mitigating or possibly eliminating the BP-lowering effects seen with systemic delivery. For future studies of ARB delivery with eye drops, our LC/MS methods for measuring plasma and eye concentrations of ARBs will allow us to verify delivery to the eye and to test for low systemic concentrations.

This study has several limitations. First, mice were followed for a relatively short period. Since interference with TGFβ signaling should affect extracellular matrix turnover, it is possible that longer term treatments could lead to remodeling of the trabecular meshwork, which may have a greater impact on IOP. In addition, IOP was determined in the daytime. Nocturnal IOP is higher in mice. This may have resulted in a greater relative reduction of IOP by ARBs that may have been missed, particularly for losartan. Also, because of the variable pSmad2 expression in the trabecular meshwork, we were not able to draw conclusions regarding TGFβ signaling in the trabecular meshwork, which is relevant to IOP regulation. Another limitation is that we measured ARB concentration in the whole eye. Although we detected relevant concentrations of ARBs, this was at the lower end of our detectable range, and therefore we could not investigate drug concentration in relevant tissues such as the trabecular meshwork, optic nerve, or in the aqueous humor. The whole eye includes the highly vascularized choroid, so to some extent whole eye drug levels are a reflection of systemic concentrations measured in plasma. But, we did detect ARBs in the cortex of the brain, which is separated from systemic circulation by the blood-brain barrier. Thus, our data clearly show that ARBs effectively cross the blood-brain barrier, which is a somewhat controversial topic in pharmacology of ARBs. Presumably, ARBs would also be able to cross the blood-aqueous and blood-retinal barriers since these are physiologically similar structures to the blood-brain barrier.

Our results comparing losartan, irbesartan and telmisartan indicate divergent properties of ARBs that may be significant in the context of treating glaucoma. We have established that delivery of these largely hydrophobic drugs by incorporation into chow results in physiologically relevant doses, facilitating studies using rodent models of glaucoma to compare effects of different ARBs. Future studies will compare effectiveness of ARBs in mouse glaucoma models and will investigate ARBs formulated as eye drops.
